# Inhibition of Macrophage Migration Inhibitory Factor Protects against Inflammation through a Toll-like Receptor-Related Pathway after Diffuse Axonal Injury in Rats

**DOI:** 10.1155/2020/5946205

**Published:** 2020-09-05

**Authors:** Yonglin Zhao, Xing Wei, Weimiao Li, Changyou Shan, Jinning Song, Ming Zhang

**Affiliations:** ^1^Department of Oncology, Second Affiliated Hospital of Xi'an Jiaotong University, Xi'an 710004, China; ^2^Department of Gynecology and Obstetrics, Second Affiliated Hospital of Xi'an Jiaotong University, Xi'an 710004, China; ^3^Department of Neurosurgery, First Affiliated Hospital of Xi'an Jiaotong University, Xi'an 710061, China; ^4^Department of Neurosurgery, Second Affiliated Hospital of Xi'an Jiaotong University, No. 157 Xiwu Road, Xi'an 710004, China

## Abstract

**Objective:**

We have previously demonstrated that inflammation induced by toll-like receptors (TLRs) 2/4 exert cerebral deleterious effects after diffuse axonal injury (DAI); however, the underlying mechanisms are not fully understood. Macrophage migration inhibitory factor (MIF) is a multifunctional cytokine involved in inflammatory responses. The purpose of this study was to investigate the role of MIF in inflammation induced by TLRs in the cortices of DAI rats.

**Methods:**

The rat DAI model was established by head rotational acceleration and confirmed by *β*-APP, HE, and silver staining. MIF protein expression at 3 h, 6 h, 12 h, 1 d, and 3 d after DAI was measured by western blot. The localization of MIF was measured by immunofluorescence. MIF antagonist ISO-1 was intracerebroventricularly injected to inhibit MIF. Neuronal and axonal injury and glial responses were assessed by TUNEL, immunohistochemistry, and TEM. Expression of TLR2, TLR4, ERK, phospho-ERK, NF-*κ*B, and phospho-NF-*κ*B was examined by western blot. The level of IL-1*β*, IL-6, and TNF-*α* was measured by ELISA.

**Results:**

MIF expression was significantly increased, peaking at 1 day after DAI, and MIF was mainly localized in microglial cells and neurons. ISO-1 suppressed neuronal apoptosis, axonal injury, and glial responses and decreased the expression of downstream signaling molecules related to TLR2/4, including ERK, phospho-ERK, NF-*κ*B, phospho-NF-*κ*B, IL-1*β*, IL-6, and TNF-*α*.

**Conclusion:**

MIF was involved in the neuronal and axonal damage through a TLR-related pathway following DAI.

## 1. Introduction

Diffuse axonal injury (DAI), caused by shearing forces leading to widespread tearing of axons, is considered an important pathologic features of traumatic brain injury. Dysregulated neuroinflammation exacerbates secondary injury following DAI [1]. We previously found that toll-like receptors (TLRs) 2 and 4, localized primarily in microglia, might be involved in mediating secondary injury after DAI by inducing inflammation via MAPK or NF-*κ*B pathways [2, 3]. High-mobility group box 1 (HMGB1), considered as stress-released alarmins, are chromatin-associated protein that can initiate a noninfectious inflammatory response through TLR2/4 pathway [4]. Our previous study found that HMGB1 may be involved in the inflammatory response after DAI [5]. However, whether there are other factors that could initiate inflammatory response through TLR2/4 pathway after DAI is still not clear.

Macrophage migration inhibitory factor (MIF), mainly expressed in macrophages, monocytes, and endothelial cells, is a multifunctional cytokine that participates in inflammatory responses. Increased expression and secretion of MIF exerts a proinflammatory action in both local and systemic inflammatory responses through TLR pathway [6]. Blocking the activity of MIF by its antagonist 3-(4-hydroxyphenyl)-4,5-dihydro-5-isoxazole acetic acid methyl ester (ISO-1) could reduce the inflammation in acute otitis media mice in which process TLR-4 and NF-*κ*B were involved [7]. TLR2 gene expression was also significantly increased following recombinant type II MIF homologue treatment [8]. Whether MIF is involved in the initiation of TLR2/4 pathway after DAI is unknown.

The purpose of this study was to investigate the role of MIF in inflammation induced by TLRs in the cortices of DAI rats. This study elucidated the dynamic expression and localization of MIF after DAI, assessed the effects of MIF inhibition on apoptosis, axonal injury, and glial response in the cortex after injury, and investigated the relationship between MIF and TLRs.

## 2. Methods

### 2.1. Animals and Groups

Sprague–Dawley rats (*n* = 100, weighing 250–300 g, 8–10 weeks old) were supplied by the Experimental Animal Center of Xi'an Jiaotong University (License no. SCXK (Shanxi) 2006-001). Animals were housed and fed in a standard environment with a 24 h light–dark cycle at 23–26°C. All procedures were carried out in accordance with the USA National Institutes of Health “Guidelines for the Care and Use of Laboratory Animals.” This research was approved by the Biomedical Ethics Committee of Animal Experiments of Shaanxi Province in China (2018-145). The rats were divided randomly into seven groups by using random number tables: control (*n* = 20), DAI 3 h (*n* = 10), DAI 6 h (*n* = 10), DAI 12 h (*n* = 10), DAI 1 d (*n* = 20), DAI 3 d (*n* = 10), and DAI 1 d + ISO-1 (*n* = 20). The DAI 1 d + ISO-1 group was injected intracerebroventricularly with 10 *μ*L of the ISO-1 (1 mg/kg, 5052250001, Sigma-Aldrich, USA) mixture 0.5 h before modeling [9]. The mixture was injected 2.3 mm posterior to the bregma, 1.5 mm lateral to the midline, and 3.5 mm deep to the cerebral dura mater surface into the right lateral ventricle using a microsyringe under the guidance of a stereotaxic apparatus.

### 2.2. DAI Animal Model

A DAI model was established using a lateral head rotation device [2, 3]. Briefly, after anesthesia with intraperitoneal injection of 1% (*w*/*v*) pentobarbital sodium (35 mg/kg), the rat's head was secured horizontally to the lateral head rotation device, with its body at a 30° oblique angle to the top of the laboratory table. When the switch was triggered, the rat's head was rotated instantly by 90° before a sudden stop. All injured rats suffered primary coma. Rats in the control group only experienced anesthesia and fixation to the device. Five rats died from injuries in this study (3 in DAI 1 d group and 2 in DAI + ISO-1 group), were excluded, and then replaced by new rats.

### 2.3. Hematoxylin and Eosin (H&E) and Golgi Silver Staining

Tissue sections were stained with H&E and processed for Golgi staining according to the manufacturer's instructions for the FD Rapid GolgiStain™ Kit (FD NeuroTechnologies, Columbia, MD, USA). All sections were observed with a light microscope at ×40 magnification.

### 2.4. Immunohistochemical Staining and Semiquantitative Analysis

Tissue sections were subjected to histochemical staining by the streptavidin-biotin peroxidase complex method. Sections were deparaffinized and rehydrated before inactivating endogenous peroxidases and then blocked with serum after antigen unmasking. Sections were incubated with rabbit monoclonal *β*-amyloid precursor protein (*β*-APP) (1 : 300, Abcam, Cambridge, UK), mouse monoclonal glial fibrillary acidic protein (GFAP) (1 : 400, Cell Signaling Technology (CST), Danvers, MA, USA), rabbit polyclonal ionized calcium-binding adapter molecule-1 (Iba-1) (1 : 300, Wako, Tokyo, Japan), rabbit monoclonal neurofilament light chain (NF-L, 1 : 100, CST), mouse monoclonal neurofilament heavy chain (NF-H, 1 : 100, CST), or mouse monoclonal neurofilament medium chain (NF-M, 1 : 100, CST) antibodies. Immunoreactivity was scored based on the number of positive cells and staining intensity. Immunohistochemical scores were determined by multiplying the quantity and staining intensity scores as follows: (1) the quantity was rated on a scale of 0–4 with no staining, 0; 1–10% of cells stained, 1; 11–50%, 2; 51–80%, 3; and 81–100%, 4; (2) staining intensity was rated on a scale of 0–3, with 0, negative; 1, weak; 2, moderate; and 3, strong. Theoretically, the scores could range from 0 to 12. An immunohistochemical score of 9–12 was considered strongly immunoreactive, 5–8 moderate, 1–4 weak, and 0 negative [10].

### 2.5. Terminal Deoxynucleotidyl Transferase dUTP Nick-End Labeling (TUNEL) Assay

The DeadEnd Fluorometric TUNEL System (Promega, Madison, WI, USA) was used to detect apoptosis in rat cortices, as per the manufacturer's instructions. Briefly, sections were incubated with equilibration buffer and equilibrated at room temperature, followed by TdT reaction incubation. The nuclei were stained with DAPI. Fluorescence was detected by fluorescence microscopy. Data are expressed as the ratio of TUNEL stained to total nuclei.

### 2.6. Immunofluorescence Staining

Brain tissue processing methods were the same as described for immunohistochemical staining. Sections were incubated overnight with primary antibodies including rabbit polyclonal MIF (1 : 250, Abcam), mouse monoclonal NeuN (1: 400, EMD-Millipore, Billerica, MA, USA), mouse monoclonal GFAP, and goat polyclonal Iba-1 (1 : 200, Abcam). Sections were incubated with a fluorochrome-conjugated secondary antibody and 4′,6-diamidino-2-phenylindole (DAPI, 1 *μ*g/mL) and then observed under a fluorescence microscope (Molecular Devices, Sunnyvale, CA, USA).

### 2.7. Western Blotting Analyses

The methods used were the same as described previously [2, 3]. Briefly, tissues were lysed to extract the total protein. Equal amounts of total proteins were separately electrophoresed on a 12% polyacrylamide gel and then were transferred onto a polyvinylidene difluoride membrane. The membranes were incubated with primary antibodies including mouse monoclonal *β*-actin (1 : 1000, CST), rabbit monoclonal NF-*κ*B p65 (1 : 1000, CST), rabbit monoclonal phospho-NF-*κ*B p65 (Ser536, 1 : 500, CST), rabbit polyclonal MIF (1 : 500, Abcam), rabbit polyclonal TLR2 (1 : 100, Bioss, Beijing, China), rabbit polyclonal TLR4 (1 : 200, Bioss), rabbit polyclonal phospho-p44/42 MAPKs (ERK1/2) (Thr202/Tyr204, 1 : 1000, CST), and rabbit polyclonal p44/42 MAPK (1 : 1000, CST) and then with the appropriate secondary antibody (1 : 5000). The ImageJ software (National Institutes of Health, Bethesda, MD, USA) was used to quantitate the band optical density.

### 2.8. Enzyme-Linked Immunosorbent Assay (ELISA)

Cortical tissue lysates were analyzed to determine the concentrations of inflammatory factors, including tumor necrosis factor-*α* (TNF-*α*), interleukin- (IL-) 1*β*, and IL-6 using ELISA kits (R&D Systems, Minneapolis, MN, USA) according to the manufacturer's instructions. Data (pg protein) were normalized to mg of total protein.

### 2.9. Transmission Electron Microscopy (TEM)

Tissues were fixed following the manufacturer's instructions. Briefly, samples were fixed in 1% (*w*/*v*) osmium tetroxide and dehydrated by immersing them in a series of ethanol solutions of increasing concentration, followed by resin embedding in Epon 812. Thin sections were lightly counterstained with 2% (*w*/*v*) uranyl acetate and 3% (*w*/*v*) lead citrate prior to examination. All the sections were observed under TEM (H-7650, Hitachi, Tokyo, Japan).

### 2.10. Statistical Analyses

The SPSS 19.0 statistical software (IBM, Chicago, IL, US) was used for statistical analyses. All results are expressed as means ± standard deviation. A one-way analysis of variance was utilized to compare numerical data in more than two groups, followed by the least significant difference post hoc test. A *P* value less than 0.05 was considered statistically significant.

## 3. Results

### 3.1. Histopathological Changes in DAI Rats and Dynamic Expression of MIF after DAI

**Figure 1:**
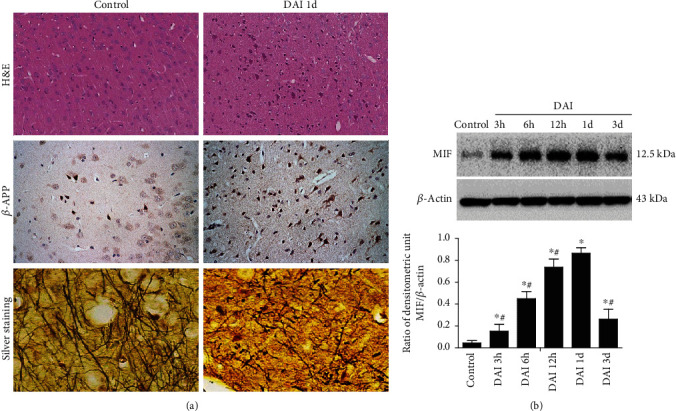
Dynamic expression of MIF in the cortex after DAI. (a) Pathological changes in DAI 1 d group and in control group were confirmed by H&E staining, *β*-APP immunohistochemistry (scale bar = 100 *μ*m), and silver stain (scale bar = 20 *μ*m). (b) Western blotting analysis was performed to measure the dynamic expression of MIF at 3, 6, and 12 h and 1 d and 3 d after DAI. Values are presented as means ± SD (*n* = 10; ∗*P* < 0.05 compared with control group; ^#^*P* < 0.05 compared with DAI 1 d group).

In H&E stained sections, vacuoles, nuclear fragmentation, and pyknosis were observed in the DAI 1 d group. Silver staining showed swollen axons and axonotmesis in this group, while in the control group, no similar abnormal histopathological changes were observed. *β*-APP expression increased significantly in the cortex of the DAI 1 d group compared with the control group (Figure 1(a)).

Western blotting was performed to examine MIF expression at different time points. Compared to the control group, MIF expression was increased significantly in DAI 3 h group, reaching a peak in DAI 1 d group, and then gradually decreasing (Figure 1(b)).

### 3.2. Localization of MIF at 1 Day after DAI

**Figure 2:**
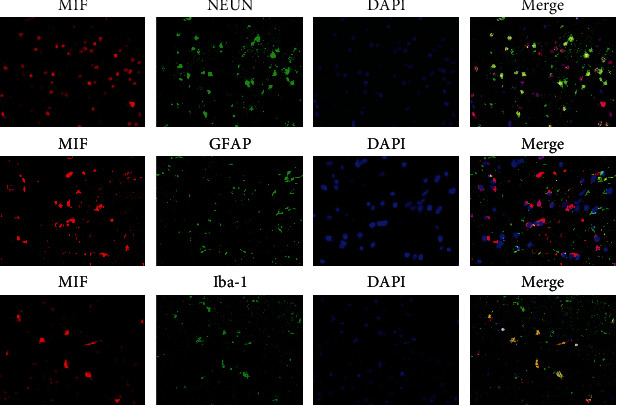
Localization of MIF in different cell types after DAI. Double immunofluorescence staining was performed for MIF (red), NeuN (marker for neurons, green), GFAP (marker for astrocytes, green), and Iba-1 (marker for microglia, green). Nuclei were stained with DAPI (blue). Scale bar = 50 *μ*m.

To determine the localization of MIF after DAI, a double immunofluorescence labeling study was performed, including NeuN (neuronal nuclei), GFAP (astrocytes), Iba-1 (microglia), and MIF. MIF- and GFAP-positive astrocytes were rarely observed in cortices of rats in the DAI 1 d group. MIF- and NeuN-positive neurons and MIF- and Iba-1-positive microglia were both obvious in the cortex of the DAI 1 d group (Figure 2). These findings suggest that increased MIF was mainly localized in microglial cells and neurons, instead of astrocytes.

### 3.3. Role of MIF on Apoptosis after DAI

**Figure 3:**
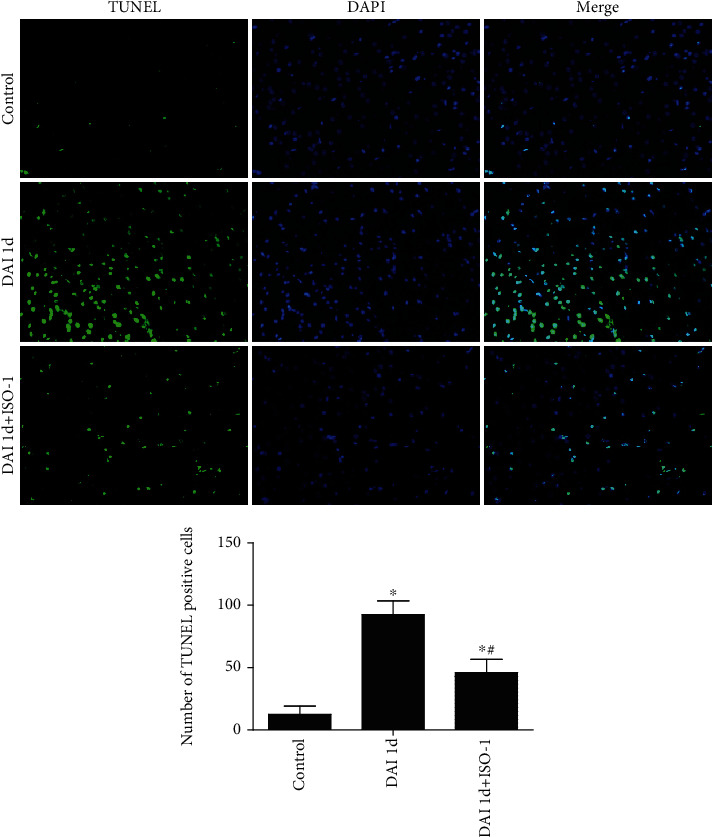
Inhibition of MIF significantly attenuates apoptosis after DAI. TUNEL assay was used to detect apoptotic cells. TUNEL-positive cells were stained green, and the nuclei were stained with DAPI (blue). Scale bar = 100 *μ*m. Bar graphs show the numbers of apoptotic cells that were counted in five random cortical fields (×40 magnification). Data are presented as means ± SD (*n* = 10; ∗*P* < 0.05 compared with the control group; ^#^*P* < 0.05 compared with DAI 1 d + ISO-1 group).

Few TUNEL-positive cells were detected in the control group. Compared to the control group, the number of TUNEL-positive cells was increased in cortices of the DAI 1 d group. Compared to the DAI 1 d group, the number of TUNEL-positive cells was significantly decreased in the DAI 1 d + ISO-1 group (Figure 3).

### 3.4. Role of MIF on Axonal Damage after DAI

**Figure 4:**
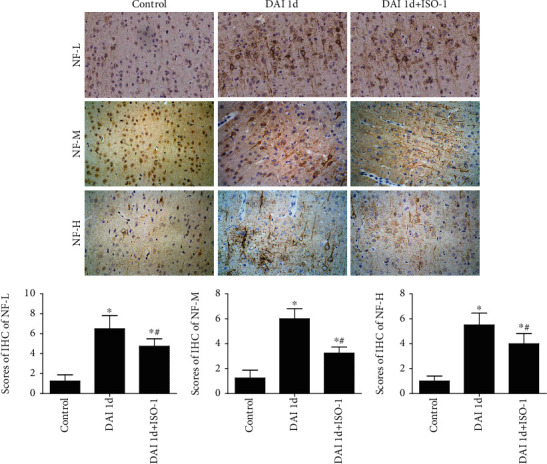
Protective effect of MIF inhibition in axons after DAI. NF-L, NF-M, and NF-H immunohistochemistry was performed to assess pathological changes. (b) Bar graphs show the NF-L, NF-M, and NF-H immunohistochemistry results. Immunoreactivity was assessed in five random fields (×40 magnification) via immunohistochemical scores. Data are presented as means ± SD (*n* = 10; ∗*P* < 0.05 compared with the control group; ^#^*P* < 0.05 compared with the DAI 1 d + ISO-1 group).

NF-L, NF-M, and NF-H were considered markers of axonal damage [2, 11, 12]. Compared to the control group, NF-L, NF-M, and NF-H expression levels were increased significantly in cortices of the DAI 1 d group. Compared to the DAI 1 d group, the expression of NF-L, NF-M, and NF-H were decreased in the DAI 1 d + ISO-1 group (Figure 4).

**Figure 5:**
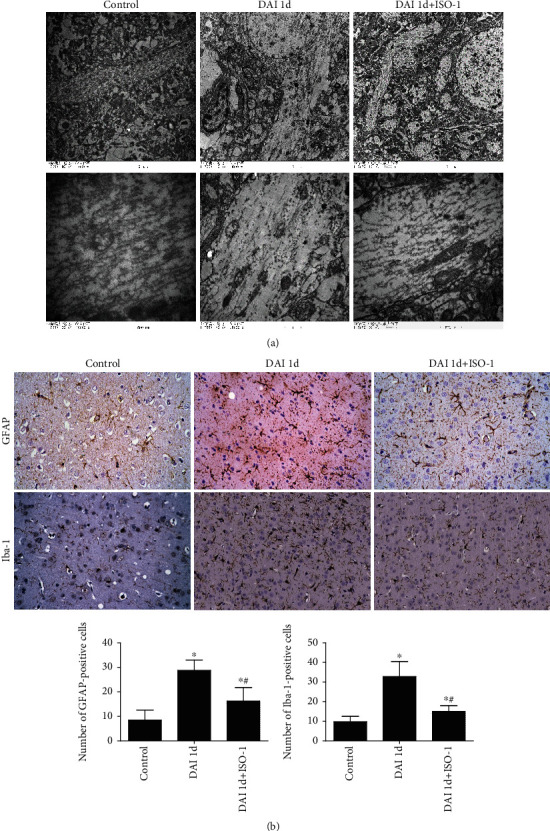
MIF inhibition attenuates glial responses and protects against ultrastructural alterations of axons. (a) Ultrastructural alterations of the longitudinal sections of axons after DAI were observed by TEM (scale bars = 5 *μ*m and 500 nm, *n* = 10). (b) GFAP and Iba-1 were assessed via immunohistochemistry in rat cortices. Scale bar = 50 *μ*m. Data are presented as means ± SD (*n* = 10; ∗*P* < 0.05 compared with the control group; ^#^*P* < 0.05 compared with the DAI 1 d + ISO-1 group).

TEM results showed that, in the control group, normal microtubules were consecutive, integrated, and compact. In the DAI 1 d group, the normal structure of microtubules was damaged, frayed, and disappeared. Furthermore, microtubules displayed conspicuous free ends. In the DAI 1 d + ISO-1 group, the structure of microtubules was partially preserved, and axon continuity was protected; even normal neurons could be seen (Figure 5(a)).

### 3.5. Role of MIF in Glial Responses after DAI

Compared to the control group, astrocyte and microglial cell staining was more robust, and the number of Iba-1-positive microglial cells and GFAP-positive astrocytes in the cortex was significantly increased in the DAI 1 d group. Compared to the DAI 1 d group, staining and numbers of Iba-1-positive microglia and GFAP-positive astrocytes were significantly decreased in the DAI 1 d + ISO-1 group (Figure 5(b)).

### 3.6. Protein Expression Related to the TLR4/NF-*κ*B and TLR2/ERK Signaling Pathways after DAI

**Figure 6:**
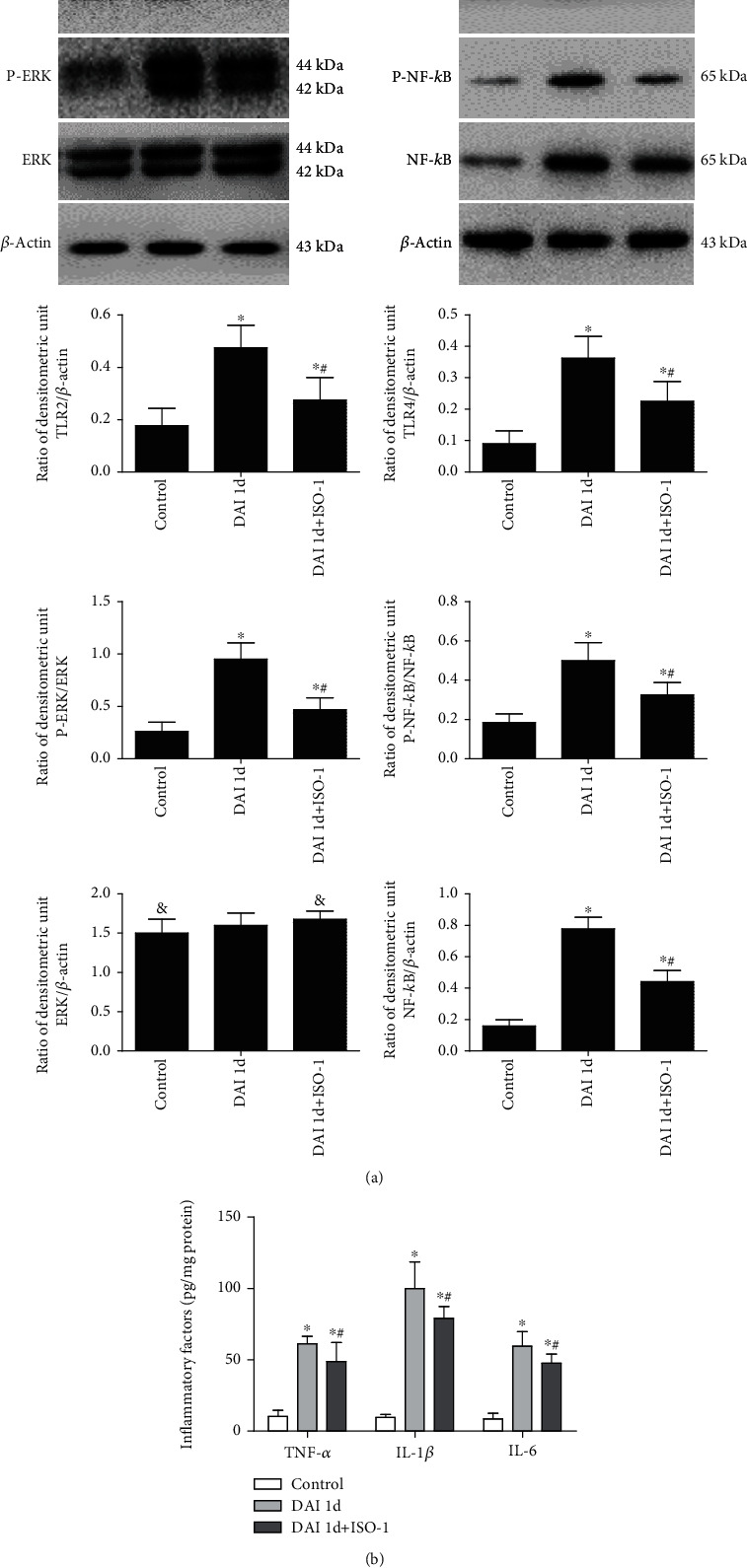
TLR2/4 signaling pathway-induced inflammatory factors are involved in DAI. (a) Expression and phosphorylation levels of signaling molecules including TLR2, TLR4, ERK, and NF-*κ*B in cortices were examined by western blotting. *β*-Actin expression was used as an internal control. (b) Effects of ISO-1 on the levels of inflammatory factors including TNF-*α*, IL-1*β*, and IL-6 in rat cortex after DAI were determined by ELISA. Data are presented as means ± SD from three separate experiments (*n* = 10; ∗*P* < 0.05 compared with the control group; ^#^*P* < 0.05 compared with the DAI 1 d group; ^&^*P* > 0.05 compared with the DAI 1 d group).

Western blotting analyses were performed to examine the expression of TLR2/4 signaling molecules including TLR2, TLR4, ERK, phospho-ERK, NF-*κ*B, and phospho-NF-*κ*B (Figure 6(a)). Compared to the control group, the expression of TLR2, TLR4, and NF-*κ*B, and phosphorylated ERK and NF-*κ*B in the cortex, significantly increased in the DAI 1 d group. Compared to the DAI 1 d group, expression of all the factors was decreased in the DAI 1 d + ISO-1 group. The ELISA results indicated that the levels of inflammatory factors, including TNF-*α*, IL-1*β*, and IL-6, were significantly increased in the DAI 1 d group compared to the control group. In contrast, levels of these inflammatory factors were significantly decreased in the DAI 1 d + ISO-1 group compared to the DAI 1 d group (Figure 6(b)).

## 4. Discussion

Our previous studies demonstrated that TLR2 and TLR4 were both involved in mediating secondary injury after DAI by inducing inflammation via MAPK or NF-*κ*B pathways, but the mechanisms of initiation are unknown. MIF is pleiotropic cytokine that has multiple effects in many inflammatory diseases. In this study, the expression of MIF was increased and peaked at 1 d after DAI and inhibition of MIF exhibited decreased apoptosis, axonal injury, and glial response. All results indicated that the MIF may be associated with secondary axonal injury. Meanwhile, DAI model used in this study showed that *β*-APP, NF-L, NF-M, and NF-H accumulated rapidly in areas of disrupted axon after DAI, similar to our previous study [13].

MIF expression is upregulated in many CNS diseases such as Alzheimer's, cerebral ischemia/reperfusion, neuroblastoma, and traumatic brain injury [14, 15]. However, the role of MIF in these diseases is contradictory. MIF overexpression is considered a negative prognostic factor in neuroblastoma [16]. Neuronal secretion of MIF might serve as a defense mechanism to compensate for decreased cognitive function in Alzheimer's disease, and increased MIF level could be a potential biomarker for the disease [17]. Early exercise could improve motor and neuronal recovery after ischemic stroke, and the increased level of MIF in the penumbra may be one mechanism of enhanced neurological function recovery [18]. In this study, we found MIF expression was increased significantly in the cortex 3 h after injury, reaching a peak at 1 day, and then gradually decreasing. Combined with our previous studies, the general tendency of MIF expression was in accord with the degree of axonal damage, which indicated MIF might be involved in the path pathological process after DAI [2, 3, 11].

Increased serum MIF concentrations have a close relationship to inflammation, trauma severity, and clinical outcomes, substantiating MIF as a good prognostic biomarker after traumatic brain injury [15]. MIF is significantly elevated in subjects with acute spinal cord injury and may contribute to primary and secondary functional outcomes [14]. In this study, MIF inhibition alleviated axonal injury as assessed by TEM, tau, NF-L, NF-M, and NF-H expression as assessed by western blots, decreased apoptosis as assessed by TUNEL assay, and reduced glial responses as assessed by Iba-1 and GFAP expression. Also, previous studies found MIF promoted neuronal death and aggravates neurologic deficits after experimental stroke and ISO-1 protected against cell death [19]. Overexpression of MIF exaggerates ocular inflammation, and this exaggerated inflammation is associated with the activation of the Notch signaling [20]. However, other studies have found that MIF administration exhibited neuroprotective effects. For example, MIF could promote neuronal cell survival and induced high expression levels of Bcl2 (antiapoptosis) and low expression levels of Caspase-3 and Bax (proapoptosis) in an oxygen-glucose deprivation/reperfusion model [21]. MIF can also induce neural stem/progenitor cells proliferation and maintenance for the treatment of degenerative brain disorders [22]. All the results indicated that the role of MIF in nervous system disease is still controversial and need further study.

Our results showed that the expression of TLR2 and TLR4, and phosphorylation of ERK and NF-*κ*B in the cortex, significantly increased at 1 d after DAI and decreased when MIF was inhibited. Meanwhile, levels of TNF-*α*, IL-1*β*, and IL-6 in rat cortices also significantly decreased when MIF were inhibited. Our previous study indicated that activation of TLR4 signaling led to NF-*κ*B activation and secretion of IL-6, IL-1*β*, and TNF-*α* and was associated with the TLR2-activated ERK1/2 pathway. Concentrations of these inflammatory factors were downregulated by inhibiting TLR4 and TLR2. In the process of DAI, microglia and astrocytes released proinflammatory molecules. Persistent inflammation after injury has been linked to both posttraumatic neurodegeneration and functional dysfunctions in many studies [23]. This study found MIF antagonist could inhibit the TLR2/4 pathway, same as previous studies. It has been found that blocking the activity of MIF by ISO-1 could reduce the inflammation in acute otitis media mice in which process TLR-4 and NF-*κ*B were involved [7]. It was also possible that MIF may mediate acute kidney injury via CD74/TLR4-NF-*κ*B signaling [24]. In addition, TLR2 gene expression was also significantly increased following recombinant type II MIF homologue treatment [8].

In addition, there are certain limitations in our current study, which will be addressed in further studies: (1) although we found inhibition of MIF has the neuroprotective effect on DAI, the MIF antagonist is not available for clinical application since many studies in murine models may not be extrapolated to humans. (2) MIF acts as a ligand for CD74 receptor. In other studies, the number of MIF receptor- (CD74-) positive cells within the ischemic brain hemisphere did not differ significantly between the WT and MIF-KO mice subjected to transient middle cerebral artery occlusion. In this study, we did not access the expression of MIF receptors

## 5. Conclusion

MIF expression was significantly increased, and MIF was mainly localized in microglial cells and neurons, after DAI. MIF inhibition attenuated neuronal apoptosis, axonal injury, and glial responses. Therefore, MIF may induce neuronal and axonal damage by increasing levels of IL-6, IL-1*β*, and TNF-*α* through TLR-related pathways following DAI.

## Data Availability

Some or all data, models, or code generated or used during the study are available in a repository or online in accordance with funder data retention policies (provide full citations that include URLs or DOIs.)
