# Correction to “Inhibition of Macrophage Migration Inhibitory Factor Protects against Inflammation through a Toll‐like Receptor‐Related Pathway after Diffuse Axonal Injury in Rats”

**DOI:** 10.1155/bmri/9804519

**Published:** 2026-04-08

Y. Zhao, X. Wei, W. Li, C. Shan, J. Song, and M. Zhang, “Inhibition of Macrophage Migration Inhibitory Factor Protects Against Inflammation Through a Toll‐Like Receptor‐Related Pathway After Diffuse Axonal Injury in Rats” *BioMed Research International*, No. 2020 (2020). https://doi.org/10.1155/2020/5946205.

In the article titled “Inhibition of Macrophage Migration Inhibitory Factor Protects against Inflammation through a Toll‐like Receptor‐Related Pathway after Diffuse Axonal Injury in Rats”, there was an error in Figure 1a related to an incorrect image of silver‐stained cortical tissue from the control group. The error was introduced by the authors during figure assembly and Figure 1 should be corrected as follows:

Figure 1Dynamic expression of MIF in the cortex after DAI. (a) Pathological changes in DAI 1‐day group and in control group were confirmed by H&E staining, *β*‐APP immunohistochemistry (scale bar = 100 *μ*m), and silver stain (scale bar = 20 *μ*m). (b) Western blotting analysis was performed to measure the dynamic expression of MIF at 3, 6, and 12 h and 1 and 3 days after DAI. Values are presented as means ± SD (n = 10;  ^∗^p < 0.05 compared with control group; #*p* < 0.05 compared with DAI 1‐day group). 3 BioMed Research International.

**Figure:**
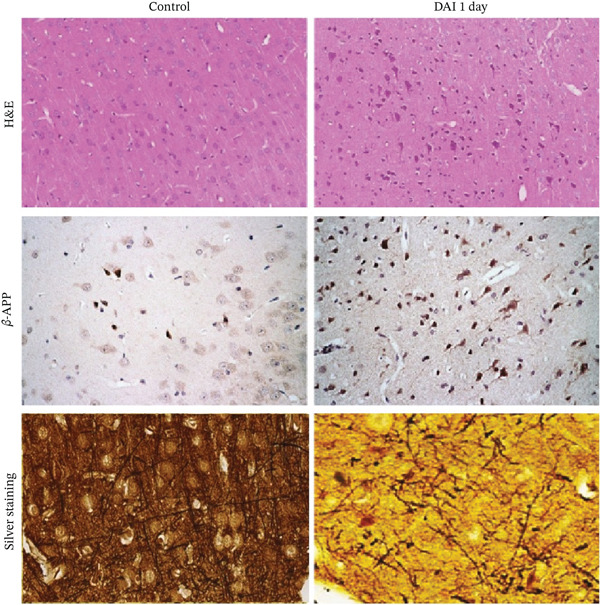
(a)

**Figure:**
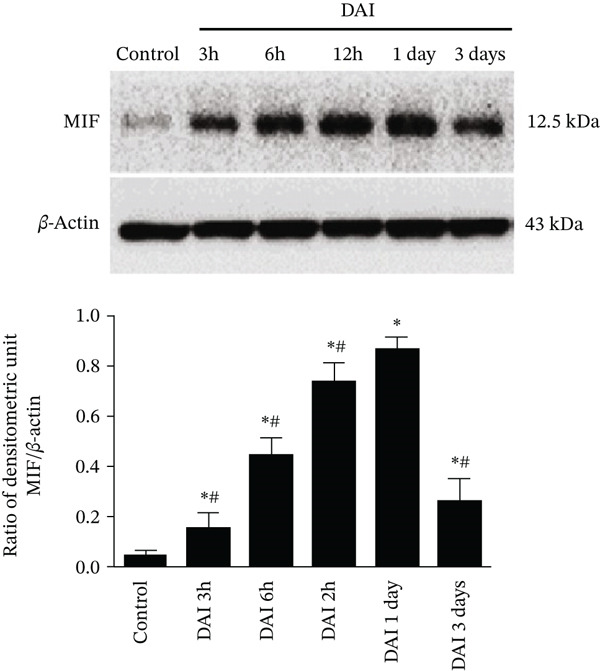
(b)

We apologize for this error.

